# Characterization and phylogenetic analysis of the mitochondrial genome of *xanthabraxas hemionata* (lepidoptera: Geometridae)

**DOI:** 10.1080/23802359.2022.2107447

**Published:** 2022-08-17

**Authors:** Hongyu Chen, Wei Li, Shuo Shen

**Affiliations:** a Academy of Agriculture and Forestry Sciences, Qinghai University, Xining, China; b State Key Laboratory of Plateau Ecology and Agriculture, Qinghai University, Xining, China; c Ministry of Agriculture, Scientific Observing and Experimental Station of Crop Pest in Xining, Xining, China; d Key Laboratory of Agricultural Integrated Pest Management of Qinghai Province, Xining, China

**Keywords:** Geometridae, *Xanthabraxas hemionata*, mitochondrial genome, phylogenetic analysis

## Abstract

*Xanthabraxas hemionata* (Guenée, 1858) is a medium-sized and slender-bodied forest pest in China. In this study, we sequenced and analyzed the complete mitochondrial genome (mitogenome) of *X. hemionata*. This mitogenome was 15,309 bp long and encoded 13 protein-coding genes (PCGs), 22 transfer RNA genes (tRNAs), and 2 ribosomal RNA genes (rRNAs). The gene order was conserved and identical to that of most other previously sequenced Geometridae species. Most of the PCGs of *X. hemionata*, except for *cox1,* have the conventional ATN start codon. Except for four genes (*cox1*, *cox2*, *nad5,* and *nad4*) that end with the incomplete stop codon T−, all other PCGs terminated with the stop codon TAA. Phylogenetic analysis positioned *X. hemionata* in a well-supported clade with the other Ennominae species. The relationships (Ennominae + (Sterrhinae + Larentiinae)) were supported by Geometridae.

Geometridae is the second-most species-rich family of Lepidoptera, with approximately 24,000 described species (Van Nieukerken et al. 2011). Eight subfamilies (Sterrhinae, Larentiinae, Desmobathrinae, Archiearinae, Oenochrominae, Geometrinae, Orthostixinae, and Ennominae) are currently recognized in the Geometridae (Murillo-Ramos et al. 2019). The adults of Geometridae are generally slender-bodied with large wings. The larvae of Geometridae have a typical looping movement because the ventral prolegs of segments A3–A5 are usually absent or vestigial, and some have become infamous pest species in orchards and monoculture forest plantations (Axmacher et al. 2009; Sihvonen et al. 2011). Most are nocturnal and cryptically patterned, but several lineages include brightly-colored diurnal species. Many species of the tropical taxa of Geometridae remain undescribed, and their classification has many uncertainties (Murillo-Ramos et al. 2019). One of its species, *Xanthabraxas hemionata* (Guenée, 1858), mainly distributed in China, is a forest pest with a black spot on its bright yellow wing. To provide molecular data for further study on the phylogeny of Geometridae, we sequenced the complete mitogenome of *X. hemionata* and analyzed the phylogenetic relationships based on mitogenome data.

Male adults of *X. hemionata* were collected from Taihe City, Jiangxi Province, China (26°46′N, 114°45′E, July 2020) and stored at the Entomological Museum of Qinghai University (Accession number QHU-EXH05, Dr. Wei Li, lwbabylw@163.com). All animal handling and experimental procedures were approved by the Animal Welfare Ethical Committee and Animal Experimental Ethics Committee of Qinghai University (Xining, China). Total genomic DNA was extracted from the muscle tissues of the thorax using a DNeasy DNA Extraction Kit (Qiagen, Hilden, Germany). A paired-end sequence library was constructed and sequenced using the Illumina HiSeq 2500 platform (Illumina, San Diego, CA, with a 150 bp pair-end sequencing method. A total of 9.0 million reads were generated and deposited in the NCBI Sequence Read Archive (SRA) under accession number SRR16518225. Raw reads were assembled using MITObim v 1.7 software with default parameters (Hahn et al. 2013). By comparison with the homologous sequences of other Geometridae species from GenBank, 13 protein-coding genes, 2 rRNA, and 22 tRNA genes of *X. hemionata* were annotated using GENEIOUS R11 (Biomatters Ltd., Auckland, New Zealand).

The complete mitogenome of *X. hemionata* is 15,309 bp in length (GenBank accession no. OK509075) and contains a typical set of 13 protein-coding genes, 2 rRNA, and 22 tRNA genes, and 1 non-coding AT-rich region. The gene order is conserved and identical to most previously sequenced Geometridae species (Liu et al. 2014; Cheng et al. 2017; Chen et al. 2019; Ding et al. 2020; Song et al. 2019). The nucleotide composition of the mitogenome was 82.6% A + T content (A 41.5%, T 41.1%, C 10.2%, G 7.2%). Four PCGs (*nad4*, *nad4l*, *nad5,* and *nad1*) were encoded by the minority strand (N-strand), whereas the other nine were located on the majority strand (J-strand). All the PCGs of *X. hemionata,* except for *cox1,* have a conventional start codon for invertebrate mitochondrial PCGs (ATN). Except for four genes (*cox1*, *cox2*, *nad5,* and *nad4*) that end with the incomplete stop codon T−, all other PCGs terminated with the stop codon TAA. The 22 tRNA genes varied in size from 64 bp (*trnR*) to 71 bp (*trnK*). Two rRNA genes (*rrnL* and *rrnS*) were located in *trnL1*/*trnV* and *trnV*/control regions, respectively. The lengths of *rrnL* and *rrnS* in *X. hemionata* were 1,405 and 771 bp, respectively, with AT contents of 85.7% and 86.5%, respectively.

Phylogenetic analysis was performed based on nucleotide sequences of 13 PCGs from 20 Geometroidea species. Alignments of individual genes were concatenated using SequenceMatrix 1.7.8 (Vaidya et al. 2011). A phylogenetic tree was constructed using raxmlGUI 1.5 (Silvestro and Michalak 2012). Phylogenetic analysis positioned *X. hemionata* in a well-supported clade with other Ennominae species (Figure 1). All 15 Ennominae species formed one clade, and the relationships (*Xanthabraxas* + ((*Phthonandria* + *Celenna*) + ((*Abraxas* + *Semiothisa*) + (*Ectropis* + (*Biston* + (*Hypomecis* + (*Erannis* + *Apocheima*)))))))) were supported. The relationships (Ennominae + (Sterrhinae + Larentiinae)) were supported in Geometridae, and similar results were found in previous studies (Sihvonen et al. 2011; Murillo-Ramos et al. 2019). These results provide an important basis for further studies on the mitochondrial genome and phylogenetics of the Geometridae.

**Figure 1.**
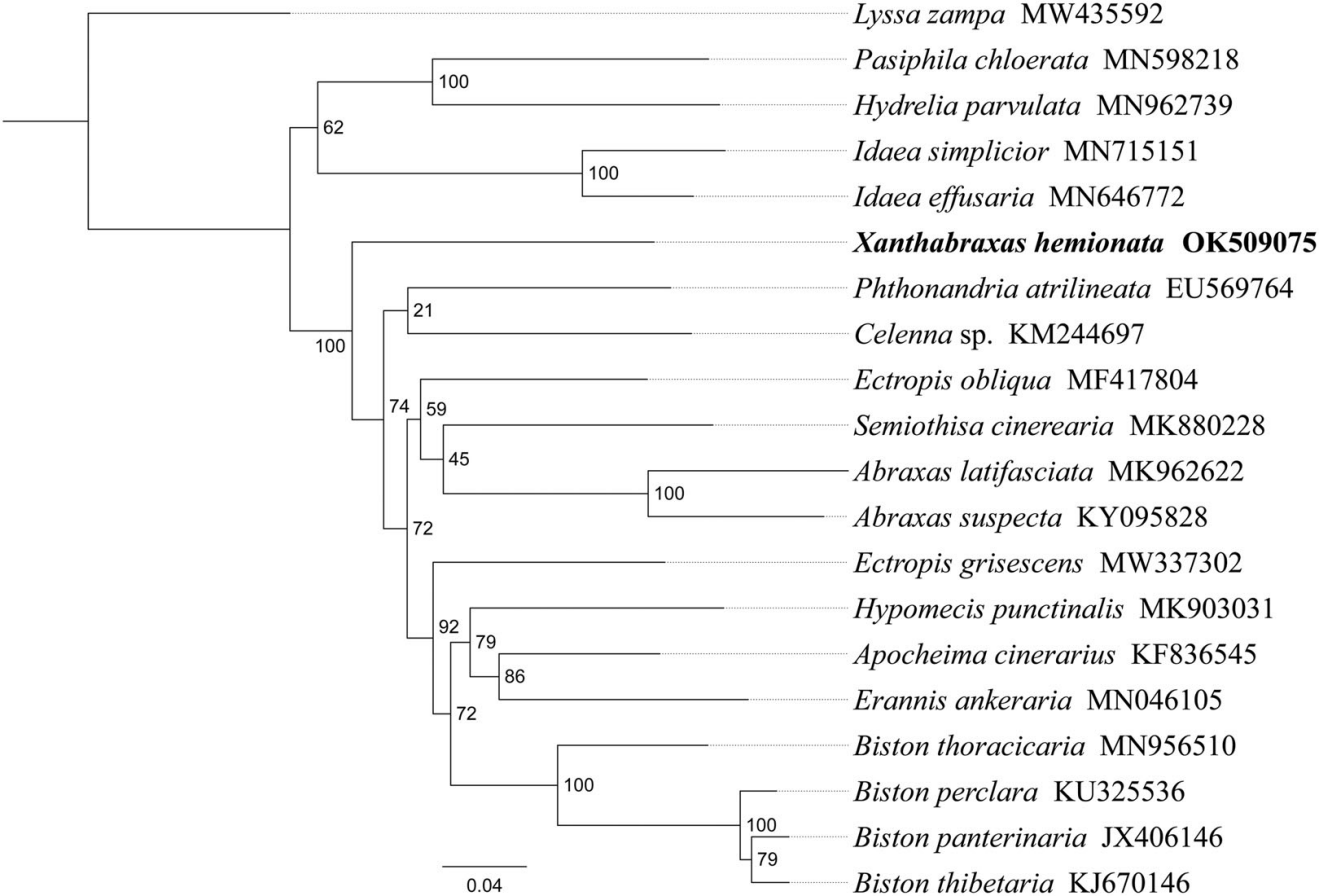
Phylogenetic tree based on the 13 mitochondrial protein-coding genes sequences inferred from Bayesian. Numbers on branches are posterior probabilities (PP).

## Data Availability

The data that support the findings of this study are openly available in NCBI (National Center for Biotechnology Information) at https://www.ncbi.nlm.nih.gov/, reference number OK509075. The associated BioProject, SRA, and Bio-Sample numbers are PRJNA770109, SRR16518225, and SAMN22208877 respectively.
